# Assessing amino acid solubility of black soldier fly larvae meal in Atlantic salmon (*Salmo salar*) *in vivo* and *in vitro*

**DOI:** 10.3389/fphys.2022.1028992

**Published:** 2022-11-23

**Authors:** Gopika Radhakrishnan, Marta S. Silva, Erik-Jan Lock, Ikram Belghit, Antony Jesu Prabhu Philip

**Affiliations:** ^1^ Institute of Marine Research, Bergen, Norway; ^2^ Department of Biological Sciences, University of Bergen, Bergen, Norway

**Keywords:** aquafeed, protein digestibility, *in vitro* assay, pH Stat, insect meal, Atlantic salmon

## Abstract

*In vitro* and *in vivo* methods were used to evaluate amino acids solubility of black soldier fly (BSF) larvae meal and two experimental diets (reference and test diets) for Atlantic salmon. The current study used *in vitro* method such as pH stat to compare and standardise the salmon extracted enzyme (SE), and commercial enzyme (CE) based on their hydrolytic capacity on a purified protein substrate. Further, an *in vitro* amino acid solubility of feed ingredients and diets were measured using the standardised enzyme volume from SE and CE. Results showed that SE and CE exhibit similar protein hydrolytic capacity upon standardisation on purified substrates. However, when using the two-stage hydrolysis (acidic and alkaline steps), significantly higher amino acid solubility was observed with CE except for glycine, and proline which were equally solubilised by both SE, and CE. No significant difference was observed between reference and test diet using the SE except for tyrosine, valine, leucine, and phenylalanine, which were significantly higher solubilised in reference diet than test diet. Whereas higher solubility of valine, isoleucine, aspartic acid, and glutamic acid was observed in test diet using CE than SE. Similarly, the solubility of valine, isoleucine, and glutamic acid were higher in BSF larvae meal when CE was used. The *in vivo* true protein digestibility of BSF larvae meal was 99%, and 81% for the test diet containing BSF larvae meal. The results demonstrated a positive correlation (r = 0.91; *p* < 0.01) between salmon and commercial enzymes but overall, no significant correlation was observed for amino acid solubility between *in vivo* and *in vitro*. However, there was a strong positive correlation for protein solubility using SE (r = 0.98) than CE (r = 0.74) with the *in vivo* true protein digestibility. The efficiency of SE, and CE can be compared, and standardised based on DH%, and hence correlates better with the *in vivo* protein digestibility but not with amino acid solubilities.

## Introduction

Marine-based ingredients are a major protein source in the diet of farmed Atlantic salmon. However, due to the growing concerns about economic and environmental sustainability of marine-based ingredients, plant-based ingredients have gradually replaced them in Atlantic salmon feeds, decreasing from ∼90% in 1990 to ∼25% in 2016 (Aas et al., 2019). However, plant-based ingredients require large amount of land and water, and have significant amount of antinutritional factors, and indigestible fibres (Kokou and Fountoulaki, 2018). Because protein is the most expensive, and a significant nutrient to be replaced, numerous research on alternative protein sources were undertaken (Glencross, 2020; Wang et al., 2021). As a result, novel sources of protein such as algae, yeast, and insects were introduced in aquafeed (Albrektsen et al., 2022). Among these, insect meal has received widespread interest due to its low carbon footprint (Huis, 2013), favourable nutritional profile, and high protein digestibility (Rodríguez-Rodríguez et al., 2022).

The European Union (EU) has approved the use of insects in animal feed, including crickets, yellow meal worms, black soldier flies, and maggots [Commission Regulation (EU) 2017/893]. Among these, black soldier fly (BSF) larvae are considered as one of the potential species because of its high growth rate, short rearing period, and well-balanced amino acid profile (Nogales-Mérida et al., 2019). Thus, BSF larvae meal has been used as a protein source in the salmonid diets (Belghit et al., 2019abib_belghit_et_al_2019a; English et al., 2021; Fisher et al., 2020; Weththasinghe et al., 2021) exhibiting positive (Randazzo et al., 2021), negative (St-Hilaire et al., 2007; Kroeckel et al., 2012; Gasco et al., 2016), or no effect on growth performance and nutrient digestibility (Lock et al., 2016; Dumas et al., 2018; Belghit et al., 2019b; Weththasinghe et al., 2021). However, these findings display an inconsistent result as discussed by English et al. (2021) mainly due to inter-study variations and could be linked to their digestibility. Hence, more research is required to investigate the digestibility of BSF larvae meal in the fish diet.

Nutrient digestibility is typically done *in vivo* by conducting a feeding trial. However, the feeding trial requires the use of many experimental fish, demands time, and labour (Tibbetts, 2012; Lewis et al., 2019). The EU has implemented legal obligations to put the 3Rs concepts of replacement, reduction, and refinement (Russell and Burch, 1959) into practice (Directive 2010/63/EU). In this scenario, *in vitro* approaches can be regarded as complementary research methods to study nutrient digestibility. The *in vitro* solubility method is in line with the 3Rs principles for assessing the digestibility of ingredients and diets in the laboratory employing simulated gut conditions and enzymes (Wang et al., 2021). A significant number of *in vitro* studies with varied methodologies were used for the nutritional evaluation of different feed ingredients using purified commercial proteases and enzymes extracted from fish (Hsu et al., 1977; Grabner, 1985; Grabner and Hofer, 1985; Eggum et al., 1989; Rahmah et al., 2016; Mcdonough et al., 2020; Rahmah et al., 2020). The current study used commercial proteases and enzyme extracted from Atlantic salmon with equal concentration. In previous studies, these proteases are standardised based on their specific enzymatic activity (CHONG et al., 2002). However, it was observed in the study by Yasumaru *et al.* (2014) that standardisation of enzymes for *in vitro* studies is more accurate by measuring the hydrolytic capacity (DH%) of enzyme. Considering these research background, a comparison between *in vitro* and *in vivo* nutrient digestibility sheds a light on the reliability of *in vitro* in predicting the nutrient digestibility. Also, emphasis on using the enzyme extracted from salmon is recommended to have a more realistic data on salmon physiology. However, commercial enzymes are most widely used for a rapid analysis. Hence, its comparison with the commercial enzyme source will enable to understand the differences in the enzyme properties. Therefore, the aim of the current study was 1) to standardise salmon extracted enzyme (SE) and commercial enzyme (CE) based on DH% and to compare the digestibility pattern and catalytic efficiency between them. 2) to carry out *in vitro* solubility using two stage hydrolysis where after the alkaline hydrolysis, the digested products are recovered for evaluating the amino acid solubility. 3) comparison between *in vivo* and *in vitro* protein digestibility and amino acid solubility to understand the complementarity of *in vitro* methods in predicting the solubility of aquafeed ingredients.

## Materials and methods

### Chemicals and reagents

Analytical reagent grade chemicals and Milli-Q^®^ water (18.2 MΩ cm) (EMD Millipore Corporation, Billerica, MA, United States) were used unless otherwise stated. Tris-HCl (GE Health care), Trichloroacetic acid (C_2_HCl_3_O_2_, Merck Life Science AS), Hemogloblin from bovine blood (95% crude protein), and casein from bovine milk (90% crude protein) from Sigma Aldrich, St. Louis, MO, United States. Sodium hydroxide (NaOH, Emsure^®^ ACS, ISO), hydrochloric acid (HCl, Emsure^®^ ACS, ISO, 37% w/w) and hydrogen peroxide (H_2_O_2_, Emsure^®^ ACS, ISO, 30% w/w) were purchased from Merck (Darmstadt, Germany). Pepsin (2500U, P7012, from porcine gastric mucosa), trypsin (5000U, T0303, from porcine pancreas) were obtained from Sigma Aldrich (St. Louis, MO, United States), and Chymotrypsin (≥40 U CAS Number 9004-07-3, from bovine pancreas) was purchased from Sigma Aldrich (St. Louis, MO, United States), and protease (11U, PT377-1G, from porcine pancreas) was purchased from Elastin Products Company (Owensville, MO, United States).

### Test ingredient and experimental diets

The test ingredient, BSF larvae meal (53% crude protein, and 13% crude lipid) was procured from Protix Biosystems BV (Dongen, Netherlands) and the experimental diets (test diet, and reference diet) were produced at Nofima’s Aquaculture Technology Center, Bergen, Norway. The fish trial was performed in Nofima’s tank facilities in Sunndalsøra, Norway. Formulation and proximate composition of the experimental diets (3-mm pellet) are as given in Table 1. The chemical composition and amino acids profile of BSF larvae meal along with traditional protein sources used in aquafeed, such as fish meal and soy protein concentrate are provided in Table 2. Yttrium oxide (0.05%) was used as an inert digestibility marker and added to the experimental diets. The test diet was made by mixing the reference diet with BSF larvae meal at 80:20 ratio. These ingredients (BSF larvae meal) and experimental diets (reference and test diets) were used for *in vitro* and *in vivo* studies.

**TABLE 1:** Formulation, and chemical composition of experimental diets fed to Atlantic salmon.

	References diet	Test diet
Ingredients (g/100 g)		
BSF meal^1^	-	20.00
Fish meal^2^	30.00	24.00
SPC^3^	17.00	13.60
Wheat gluten^4^	15.00	12.00
Wheat^5^	10.15	8.12
Fish oil^2^	15.00	12.00
Rapeseed oil^6^	5.5	4.40
Lecithin. Rapseed^7^	1	0.80
Vitamin PMX^8^	0.7	0.56
Mineral PMX^8^	0.7	0.56
Monosodiumphosphate^8^	2.5	2.00
L-Lysine^8^	0.7	0.56
L-Threonine^8^	0.1	0.08
DL-Methionin^8^	0.3	0.24
L-Histidine^8^	0.3	0.24
Choline chloride^8^	0.5	0.40
Carop. Pink (10% Astax)^8^	0.05	0.04
Yttrium oxide^9^	0.05	0.04
Water adjustment	0.45	0.36
Chemical composition (%)		
Moisture	7.0	7.0
Crude Protein	55.0	55.0
True Protein^10^	41.0	42.9
Crude Lipid	11.9	11.5
Carbohydrate	5.7	5.0
Ash	9.0	9.0
Amino acid composition (mg/g)		
Hyp	2.4	2.0
His	11.8	12.9
Tau	1.49	1.19
Ser	24.4	24.3
Arg	26.6	26.2
Gly	25.5	25.6
Asp	43	47
Glu	113	104
Thr	19.9	20.7
Ala	24.0	26.7
Pro	35	34
Lys	36	37
Tyr	16.2	19.6
Met	14.2	13.1
Val	22.6	24.5
Ile	20.7	21.4
Leu	37	38
Phe	23.5	23.6

^1^ Protix Biosystems BV (Dongen, Netherlands).

^2^ Pelagia, Norway.

^3^ Selecta, Brazil.

^4^ Tereos Syral, Belgium.

^5^ Norgesmøllene AS, norway.

^6^ Emmelev, Denmark.

^7^ Marvesa, Netherlands.

^8^ Vilomix, Norway.

^9^ VWR, norway.

^10^ True protein = sum of anhydrous amino acids.

BSF , black soldier fly; SPC , soy protein concentrate.

**TABLE 2:** Chemical and amino acid composition of black soldier fly larvae meal (BSF), fish meal (FM) and soy protein concentrate (SPC).

	BSF	FM^a^	SPC^a^
Chemical composition (%)			
Crude Protein	53.0	65.9	63.3
True Protein^b^	39.0	-	-
Crude Lipid	13.4	10.7	2.0
Amino acid composition (mg/g)			
His	13.5	18.2	15.5
Ser	20.9	32.6	32.4
Arg	24.1	42.3	42.9
Gly	24.2	50.3	25.6
Asp	51.0	69.2	71.3
Glu	58.0	105	114
Thr	20.8	33.3	25.1
Ala	31.0	47.2	26.3
Pro	27.8	32.1	30.9
Lys	35.0	58.8	37.0
Tyr	28.0	24.6	22.3
Met	9.5	19.6	9.0
Val	29.7	37.8	28.5
Ile	23.4	30.2	27.5
Leu	36.0	57.1	47.4
Phe	22.1	28.9	32.1

^a^ Data obtained from Leeper et al., 2022.

^b^ True protein = sum of anhydrous amino acids.

BSF , black soldier fly; FM , fish meal; SPC , soy protein concentrate.

### Standardisation of pH stat method for *in vitro* solubility using salmon gut enzymes and commercial enzymes

#### Extraction of crude salmon enzymes (SE)

The extraction of crude salmon enzyme (SE) method was developed based on principles described elsewhere (Alarcón et al., 2002; Yasumaru and Lemos, 2014; Rahmah et al., 2016). Two Atlantic salmon, weighing around 600–700 g were taken from the laboratory facility at the Institute of Marine research, Norway. The fish were fed at 8:00 in the morning with a commercial feed (Supreme Plus15, Skretting) at *ad libitum*. After 4 h, the fish were sacrificed using overdose (100 mg/L) of MS222, followed by a quick cephalic concussion. The fish were dissected to remove the stomach, pyloric ceca, and intestine. The pH of the stomach (4.9–5) and intestines (7.8–7.9) were noted before the excision. The stomach and the intestine along with pyloric caeca were thoroughly washed with cold distilled water to remove the blood stains and fat. These tissues were chopped into smaller pieces and homogenised with cold distilled water in 1:1 ratio using a tissue homogeniser (Polytron PT 2100). The homogenisation was performed in several pulses of approximately 30 s to avoid overheating, and the entire process of homogenisation was done keeping a glass beaker on ice to avoid damage to the tissue protein and enzymes. The homogenised samples were then centrifuged at 3,220 × g for 30 min at 4°C (Fisher Scientific, Eppendorf™ 5810R Centrifuges with A-4-81 Model Rotor). The collected supernatant which constituted the crude enzyme extract (SE) were stored at −80°C until further use. The total protein concentration in the stomach and intestinal extract was determined using Pierce™ 660 nm Protein Assay Reagent (ThermoFisher Scientific, Waltham, MA, United States) using BSA as protein standard (ThermoFisher Scientific).

#### Preparation of commercial enzyme (CE) stock solution

The commercial enzyme (CE) stock solution required for performing the standardisation and *in vitro* solubility were prepared by mixing equal amount of pepsin for gastric hydrolysis, and trypsin, chymotrypsin, and protease for alkaline hydrolysis. The concentration of 5 mg/ml was considered as stock solution for pepsin and intestinal proteases after performing several attempts with different concentrations based on the previous studies (Hsu et al., 1977; Dimes and Haard, 1994).

### Determination of enzyme activity

Total pepsin activity of SE and CE stock solution was assayed according to the method described by Anson and Mirsky (1932), using 2% hemoglobin solution as substrate. The assay was initiated by adding 5 ml of the substrate into the glass tubes named blank and test. All the tubes were placed at 37°C for approximately 10 min to equilibrate. This was followed by addition of 1 mL of enzyme solution into the test tubes and were placed at 37°C for 10 min to incubate. Later, the reaction was terminated by adding 10 ml of 5% trichloroacetic acid (TCA) to all tubes. One ml of respective enzyme solution was added into blank tube after adding TCA. All the tubes were mixed properly and were kept at 37°C for 5 min. The blank and test tubes were filtered using 0.45 µm syringe filter, and the absorbance were read at 280 nm (UV-VIS Spectrophotometer, Shimadzu, Model: UV-1800, United States). One unit of pepsin activity was defined as the change in absorbance of 0.001 per min at pH 2 at 37°C measured as TCA soluble products.

The total protease activity of SE and CE stock solution was measured according to Walter (1984). In this assay, the protease activity of the stock solution was measured using casein as the standard substrate. To begin with, 20 µL of enzyme solution was mixed with 0.5 ml of 0.1M Tris-HCl buffer (pH 8) at room temperature. The reaction was initiated by the addition of 0.5 ml of 1% casein and kept for 30 min. Later, the reaction was terminated by the addition of 0.5 ml of 20% TCA. The solution mix were allowed to stand for 10 min at room temperature, followed by centrifugation at 16,500 *g* for 5 min at 4°C. The absorbance of the reaction mixture was measured at 280 nm (UV-VIS Spectrophotometer, Shimadzu, Model: UV-1800). One unit of enzyme activity is defined as the 1 µg tyrosine released per min (Walter, 1984). All the measurements were carried out in duplicates.

### Crude salmon and commercial enzymes standardization using pH stat method

The crude enzyme extract from SE and CE were standardised for their hydrolytic capacity using pH stat according to (Yasumaru and Lemos, 2014) technique using automated titrators (848 Titrino plus-Metrohm AG, Switzerland). The enzyme activity which exhibited a similar degree of hydrolysis (DH%) in the standard substrates for both SE and CE were selected for *in vitro* solubility using two stage hydrolysis. The values which showed similar DH (%) using hemoglobin were 2.33 (SE) and 1.94 (CE), whereas DH (%) obtained were 5.90 (SE) and 6.17 (CE) using casein as a substrate.

The standard substrates used were analytical grade hemoglobin, and casein. One mL of CE stock solution (both gastric and intestinal enzyme mix) and 1 ml of extracted salmon enzyme (stomach and intestine extract) were used. All these enzyme solutions were serially diluted in 1:2 ratio into 5 tubes. Protein suspension mixtures of the standard substrate was prepared by dissolving 80 mg of substrate protein in distilled water (10 ml total suspension volume). The pH of hemoglobin and gastric enzymes were adjusted to 3 and pH 8 for casein and intestinal enzymes using 0.01 N HCl or 0.01 N NaOH, respectively. The protein hydrolysis assay was initiated by the addition of respective enzyme into the protein suspension for 60 min under continuous stirring. The whole reaction was maintained at room temperature (20–23°C). The hydrolyses were carried out in duplicates.

### *In vitro* solubility of black soldier fly larvae meal and diets

*In vitro* solubility of BSF larvae meal and diets were performed by two stage hydrolysis. The BSF larvae meal and the diets were sieved using a mesh to have fine particle size (˂1,000 µm) for measuring the DH% of the sample. The standardised enzyme activity was selected for gastric hydrolysis and alkaline hydrolysis. Two stage degree of hydrolysis was followed for determining the protein hydrolysis of the samples. Initially, the protein suspension (80 mg protein) of each test ingredient and pepsin were adjusted to the pH 3 with 0.01N HCl, and respective enzyme solutions were added into the mixture. The mixture was allowed to stand for 1 h at room temperature under continuous stirring. After 1 h, the pH of the solution was adjusted to pH 8 with 0.01 N NaOH, and intestinal enzymes were added. This mixture was again allowed to stand for 1 h at room temperature under continuous stirring. After the intestinal hydrolysis, soluble and insoluble fractions were separated. The insoluble fraction was immediately placed on ice to inactivate the enzyme activity. A blank was set up with same conditions without enzymes to determine the effect of autohydrolysis, and a control with only protein suspension in water. The amino acid solubility of the test samples, and blank were measured in relation to control values. All the tubes were stored at −20°C until further analysis. The non-soluble portion was analyzed for amino acid solubility using ultra performance liquid chromatography (UPLC, Waters Acquity UPLC system, Milford, MA, United States).

### *In vivo* digestibility of black soldier fly larvae meal and diets

An *in vivo* digestibility study was carried out in Atlantic salmon to evaluate the amino acid digestibility of BSF larvae meal. The feeding trial was conducted at NOFIMA Research Station at Sunndalsøra, Norway according to Norwegian (FOR-2015-06-18-761) and European legislation (Directive 2010/63/EU). Atlantic salmon used in this trial had a mean initial body weight of 83.2 ± 0.36 g. Each diet was tested in triplicate tanks (flow-through; closed system) thus the salmon were randomly distributed to 6 tanks (110 × 110 cm, 465 L water volume) contained 140 fish each. The fish were fed with the experimental diets for 30 days at *ad libitum* using automatic feeders (72 feeding per day, 1 min per meal, at 20 min intervals). Salmon were reared in seawater (flow rate at 21 L/min) with continuous light (24 h) at 11°C and oxygen saturation was maintained above 80% during the whole experimental period. To estimate the apparent nutrient digestibility of the BSF larvae meal and diets, feces were collected by manual stripping and were stored in polypropylene containers for freeze-drying. During freeze-drying samples were placed without lids in a freeze dryer at −20ºC and 0.2 mbar (FreeZone^®^ 18 L, Kansas City, Labconco). The freeze-dried feces, diets, and BSF larvae meal were analysed for amino acids concentrations using ultra-performance liquid chromatography (UPLC, Waters Acquity UPLC system, Milford, MA, United States).

### True protein calculation

True protein is calculated based on the sum of anhydrous amino acid residues, due to the presence of high concentrations of non-protein nitrogen (N) present in the BSF larvae. The protein content is often overestimated with 6.25 N-to-protein factor.

### Amino acid analysis

The amino acid analysis of the salmon diet, BSF larvae meals, feces and residue after two stage hydrolysis were carried out by an ultra-performance liquid chromatography (UPLC, Waters Acquity UPLC system, Milford, MA, United States). The quantitative determination was based on an accredited method by the Nordic Committee of Food Analysis (NMKL) and described in detail elsewhere (Espe et al., 2014; Belghit et al., 2019b). The results were integrated by Empower 3 (Waters, Milford, MA, United States). Amino acids were quantified using standards from Thermo Fisher Scientific (product number; 20,088 Rockford, IL 61105, United States).

### Formulae and calculations

The degree of hydrolysis (DH) of protein with stomach extract during standardisation was calculated based on the formula (Diermayr and Dehne, 1990 ):

DH=[(V×N)÷E]×(1÷P)×FpH×100 (1)

Where V, volume of acid consumed in the hydrolysis reaction (ml); N normality of the acid; E mass of substrate protein (g); P, number of peptide bonds cleaved (mol g protein^−1^). For proteins which amino acid composition is not determined, P is generally suggested as 8.0. F_pH_ 1.08 (correction factor).

The DH with pyloric caeca/intestine extract during standardisation was calculated according to (Adler-Nissen, 1986):

$$DH=B\times Nb\times \left(\frac{1}{\propto }\right)\times \left(\frac{1}{MP}\right)\times \left(\frac{1}{Htot}\right)\times 100$$ (2)

Where B, is the volume of alkali consumed (ml); Nb, normality of the alkali, α average degree of dissociation of the α-NHgroups (1/α = 1.50 for pH 8.0 at 25°C); MP, mass of substrate protein (g); H_tot_, total number of peptide bonds in the protein substrate [7.6–9.2 meqv g protein^−1^, according to the source of protein (Adler-Nissen, 1986)].

Amino acid (AA) solubility of final products from two stage hydrolysis during the *in vitro* tests were calculated as follows:

$$AA\;\left(\%\right)=100-\left(\;\frac{AA\;present\;in\;residue\;after\;digestion}{AA\;present\;in\;residue\;in\;control}*100\right)$$ (3)

Formulae used to determine apparent digestibility (AD) of nutrients in the diets and feed ingredients were previously described (Furukawa and Tsukahara, 1966). The apparent digestibility coefficient (ADC) of nutrients in the diets and apparent digestibility (AD) of the BSF larvae meal was calculated as follows:

$$ADC\;diets\;\left(\%\right)=100-100\left(\;\frac{Y\;diet}{Y\;feces}*\frac{N\;feces}{N\;diet}\right)$$ (4)

where Y is concentration of the inert marker (i.e., yttrium oxide) and N is the concentration of the nutrient.

$$ADC\;of\;ingredients\;\left(\%\right)=\left(\;\frac{Nut\;TD*AD\;TD-0.8*NutRD*AD\;RD}{0.2*Nut\;Ing}\right)$$ (5)

Nut_TD_ is nutrient concentration in test diet, AD_TD_ is the apparent digestibility of nutrients in test diet, Nut_RD_ is nutrient concentration in the reference diet, AD_RD_ is apparent digestibility of nutrients in the reference diet and Nut_Ing_ is the nutrient concentration in test ingredient.

### Statistical analysis

The software Statistica 13.4 (Statsoft Inc.) and GraphPad Prism (version 8.03, for Windows, GraphPad Software, La Jolla, CA, United States) were used for all statistical analysis. Data were tested for normality and homogeneity of variance using a Kolomogorov-Smirnov test and Shapiro-wilk test, respectively. Data from protein DH% from enzyme extract standardisation were transformed to arcsin (x^1/2^) before statistical analysis. Enzymes dilution (DH%) data were subjected to two way full-factorial ANOVA followed by Duncan’s Multiple Range Test at significance level of 95% using SAS 9.4 (SAS Institute Inc.) software for Windows (SAS, 2013, Institute, Cary NC). *In vitro* amino acid solubility data were subjected to two-way analysis of variance (ANOVA) with diet and enzyme effect as two factors. One-way ANOVA was performed to analyze any significant difference between diets for each enzyme, followed by Tukey’s multiple comparison. The *in vivo* digestibility data were analysed using *t*-test to compare between test diet and reference diet. For all statistical tests, *p*-values < 0.05 were considered significant, and all the results are expressed as mean ± standard deviation. Figures and graphs were obtained by using GraphPad Prism (version 8.03, for Windows, GraphPad Software, La Jolla, CA, United States).

## Results

### Standardisation of pH stat method for *in vitro* solubility using salmon gut enzymes and commercial enzymes

*In vitro* solubility for enzyme standardisation using pH stat method exhibited decreased degree of hydrolysis (DH%) on purified substrates like hemoglobin and casein upon serial dilution as shown in Table 3. One mL of SE extract from the stock solution exhibited significantly higher (*p* < 0.05) hydrolytic capacity than the equivalent volume of CE stock solution on hemoglobin. Whereas hydrolytic capacity of 1 ml of CE was significantly higher (*p* < 0.05) than the equivalent volume of SE stock solution on casein. The standardised DH values for SE on haemoglobin and casein were 2.33% and 5.95%, and CE on haemoglobin and casein were 1.94% and 6.17%, respectively (Table 3). The CE and the SE extract were standardised based on the dilution that exhibited similar DH% on the purified substrates.

**TABLE 3:** The enzyme activity, and protein hydrolytic performance (DH%) of salmon enzyme and commercial enzyme on the standard substrate, hemoglobin, and casein.

Stomach enzyme				
Dilution tubes	SE (U/ml)	SE (DH%)	CE (U/ml)	CE (DH%)
1	166.00	6.28*	156.00	1.94*
2	83.00	5.39*	78.00	1.18*
3	42.00	4.58*	39.00	0.65*
4	21.00	3.99*	20.00	0.01*
5	10.00	3.01*	10.00	0.00*
6	5.00	2.33*	5.00	0.00*
Intestine enzyme				
1	5.10	5.95*	8.20	7.70*
2	2.50	4.21*	4.10	6.17*
3	1.25	4.09*	2.05	5.39*
4	0.63	3.03*	1.03	4.65*
5	0.31	2.70*	0.51	3.96*
6	0.16	1.98*	0.26	3.50*

*In vitro* pH stat protein hydrolysis (DH%) carried out for 60 min at room temperature, with 80 mg protein substrate and 6 tubes of serially diluted (1:2) enzyme solutions with specific activity (U/ml). ‘*’ denotes statistical significance between the dilutions and were analysed through full-factorial ANOVA, followed by Duncan’s Multiple Range Test at significance level of 95%.

Total pepsin activity in the SE and CE stock solution was 166 U/ml, and 156 U/mL, respectively. The gastric enzyme activity that provided similar DH for SE and CE were 5 U/ml, and 156 U/ml, respectively (Table 3). Total protease activity in SE, and CE stock solution was 5.1 U/ml, 8.2 U/mL respectively. The intestinal enzyme activity that provided similar DH for SE and CE were 5 U/ml, and CE 4 U/ml respectively (Table 3) for studying the *in vitro* amino acid solubility in diets and BSF larvae meals.

### *In vitro* amino acid solubility of diets and black soldier fly larvae meal using standardised enzyme activity

**FIGURE 1:**
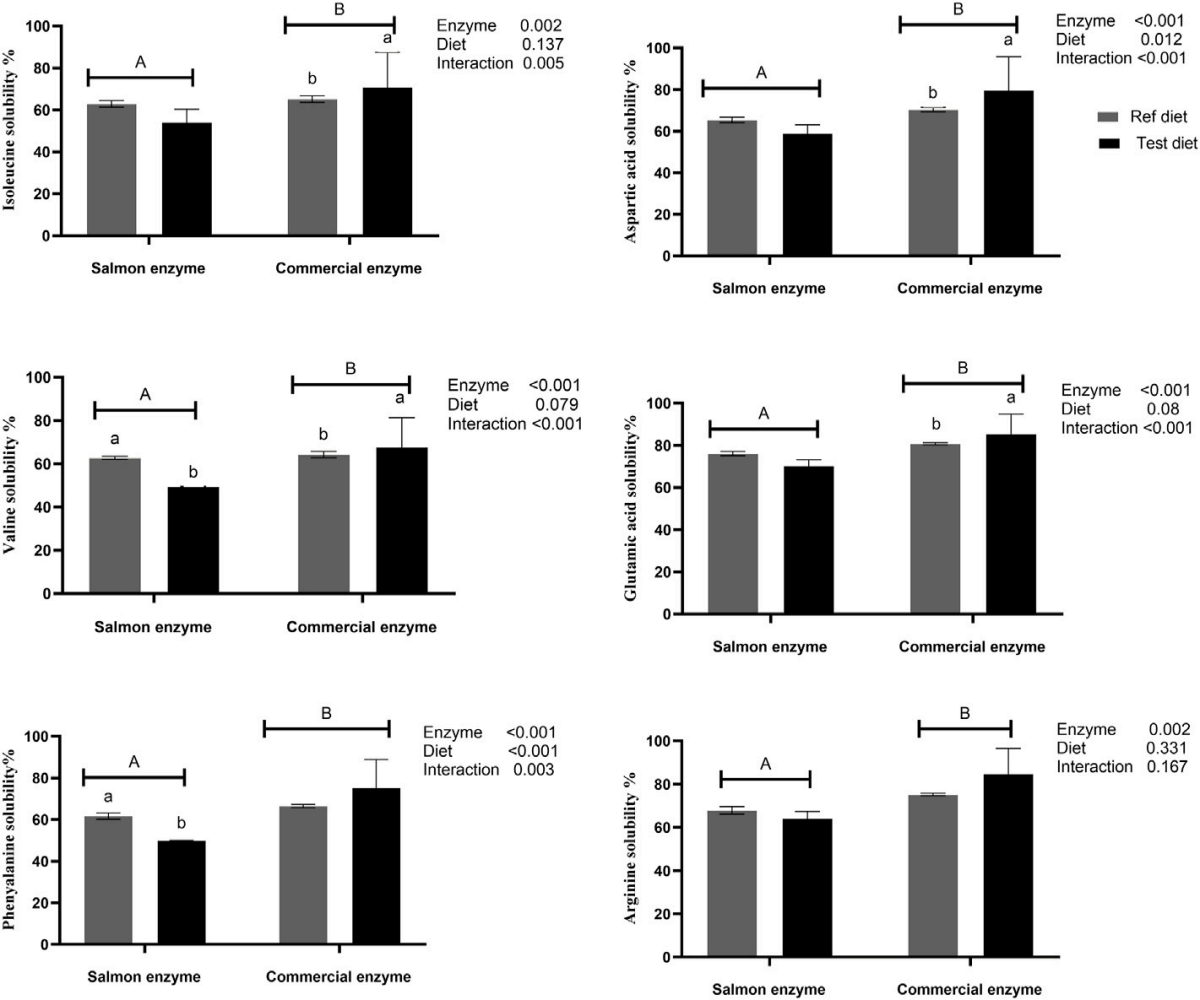
*In vitro* amino acids solubility of reference and test diets using salmon and commercial enzyme. Values are means of 2 values, with their standard deviation represented by vertical bars. Capital letters **(A,B)** indicate statistical difference between salmon enzyme and commercial enzyme detected with two-way ANOVA followed by Tukey’s multiple comparisons. Small letters (a,b) indicate the statistical difference between diets detected with one-way ANOVA. *In vitro* solubility values of the reference and test diets for remaining amino acids are presented in Supplementary Table S1.

The *in vitro* amino acid solubility of reference diet, and test diet using SE, and CE are as given in Figure 1 and Supplementary Table S1. The amino acid solubility between SE, and CE were significantly different (*p* < 0.05) despite of standardising with similar degree of protein hydrolysis (except for proline and glycine which were solubilised equally). In general, solubility of amino acids ranged between 70 and 80% using CE and 50–70% using SE, for reference diet and test diet clearly indicating the higher solubility using CE (Supplementary Table S1).

When compared between the diets using SE, there was no significant difference between test diet and reference diet except for tyrosine (*p* = 0.003), valine (*p* = 0.001), leucine (*p* = 0.049), and phenylalanine (*p* = 0.008), which exhibited significantly higher solubility in reference diet compared to test diet. Whereas significantly higher solubility was observed for aspartic acid (*p* = 0.002), glutamic acid (*p* = 0.001), valine (*p* = 0.007), and isoleucine (*p* = 0.004) in test diet when CE was used. There was a significant interaction effect between enzyme and diet on essential amino acid like valine, isoleucine, leucine, and phenylalanine, and non-essential amino acids like aspartic acid, glutamic acid, and tyrosine (Figure 1; Supplementary Table S1).

**FIGURE 2:**
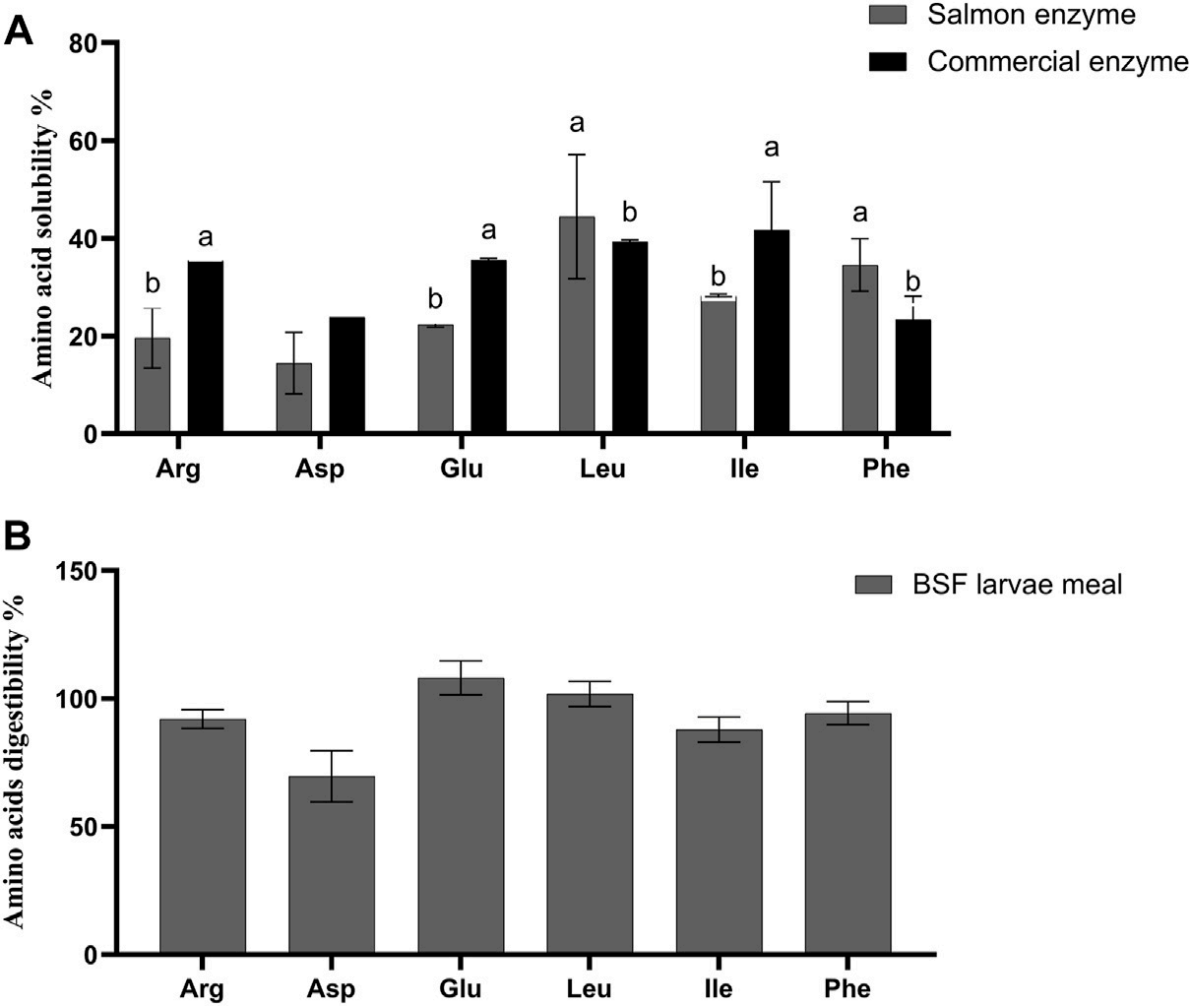
**(A)** *In vitro* amino acids solubility of black soldier fly larvae meal (BSF). Values are means of 3 values, with their standard deviation represented by vertical bars. Small letters **(A,B)** indicate the statistical difference between salmon enzyme, and commercial enzyme detected with one-way ANOVA followed by Tukey’s multiple comparisons. *In vitro* solubility values of black soldier fly larvae meal (BSF) for remaining amino acids are presented in Supplementary Table S2 **(B)** *In vivo* amino acids digestibility of black soldier fly larvae meal (BSF). Values are means of 3 values, with their standard deviation represented by vertical bars. *In vivo* digestibility values of the black soldier fly larvae meal (BSF) for remaining amino acids are presented in Supplementary Table S3.

The *in vitro* amino acid solubility of BSF larvae meal is as given in Figure 2A and Supplementary Table S2. It was observed that the solubility (%) of essential amino acid such as arginine (35.42 ± 0.31), valine (27.35 ± 3.79), isoleucine (41.73 ± 9.87) and non-essential amino acid like glutamic acid (35.53 ± 0.39), and proline (37.60 ± 1.32) were significantly higher (*p* < 0.05) solubilised with CE, being contrast to leucine (44.46 ± 12.71), phenylalanine (34.56 ± 5.38), and tyrosine (36.98 ± 13.11), which were highly solubilised with SE (*p* < 0.05). Very low solubility was observed for histidine, threonine, methionine, and aspartic acid in the BSF larvae meal with both SE and CE (Figure 2A; Supplementary Table S2).

### *In vivo* apparent digestibility of the diets and black soldier fly larvae meal

The *in vivo* apparent digestibility of true protein, crude protein, and crude lipid (%) of the diets were calculated and observed that there was no statistical difference between test diet and reference diet (Table 4). Furthermore, when compared among the amino acid digestibility, there was no significant difference between the reference diet and the test diet except for non-essential amino acids like aspartic acid (*p* = 0.009), and alanine (*p* = 0.031) which exhibited significantly higher digestibility in reference diet than test diet. The crude lipid, true protein, and crude protein digestibility (%) of the BSF larvae meal was observed to be 68.8 ± 4.4, 99.2 ± 7.3, and 89.9 ± 5.4, respectively. As given in Figure 2B, the apparent digestibility (%) of some of the amino acids such as arginine, serine, methionine, and isoleucine exhibited 92.0 ± 3.7%, 94.8 ± 6.9, 80.0 ± 6.7, and 87.98 ± 4.89, respectively.

**TABLE 4:** Apparent digestibility coefficient (ADC%) of crude lipid, true protein, crude protein and amino acid of reference and test diets fed to Atlantic salmon.

Nutrients	References diet	Test diet	*p*-value
Crude lipid	92.99 ± 0.42	93.01 ± 0.69	Ns
True protein	80.37 ± 0.46	81.19 ± 1.03	Ns
Crude protein	85.34 ± 0.34	85.63 ± 0.79	Ns
Amino acids			
His	89.14 ± 0.71	88.85 ± 0.32	Ns
Ser	87.61 ± 0.67	86.69 ± 0.67	Ns
Arg	93.05 ± 0.50	92.51 ± 0.28	Ns
Gly	81.63 ± 1.03	82.25 ± 0.85	Ns
Asp	77.69 ± 0.57a	72.28 ± 1.89 b	0.01
Glu	93.16 ± 0.42	93.03 ± 0.38	Ns
Thr	85.51 ± 0.88	84.56 ± 0.74	Ns
Ala	88.86 ± 0.58a	87.04 ± 0.77 b	0.03
Pro	90.60 ± 0.59	91.04 ± 0.47	Ns
Lys	90.61 ± 0.32	90.24 ± 0.56	Ns
Tyr	90.47 ± 0.72	89.25 ± 0.32	Ns
Met	91.45 ± 0.65	90.91 ± 0.41	Ns
Val	88.57 ± 0.86	87.57 ± 0.64	Ns
Ile	89.42 ± 0.89	88.44 ± 0.41	Ns
Leu	90.63 ± 0.65	89.90 ± 0.43	Ns
Phe	90.55 ± 0.67	89.81 ± 0.31	Ns

The values are expressed as mean ± SD (*n* = 3). Ns, not statistically significant (*p* > 0.05). Statistical significance analysed through *t* test. Different superscript letters within an individual row denote statistically significant differences in ADC, values.

### Comparison between and within *in vitro* and *in vivo* digestibility methods

A comparison was made between *in vitro* and *in vivo* true protein digestibility of BSF larvae meal (Table 5). It was observed that a strong positive correlation (r = 0.98; *p* = 0.09) was obtained using SE with the *in vivo* than using CE (r = 0.74; *p* = 0.47). No significant difference was observed for protein hydrolysis using SE, and CE on BSF larvae meal. Likewise, a comparison of amino acid solubility of BSF larvae meal using SE, CE, with *in vivo* was performed. The BSF amino acid solubility was comparatively better correlated with SE (r = 0.46; *p* = 0.07) than CE (r = 0.19; *p* = 0.46). Also, a comparison of amino acid solubility was made between diets using SE, CE, with *in vivo*. A significant positive correlation (r = 0.91; *p* < 0.01) was found between SE, and CE, in reference diet. No correlation (r = 0.26; *p* = 0.32) was found between SE, and CE, in test diet. However, when compared between *in vitro* and *in vivo,* the correlation of reference diet was found to be much lesser with SE (r = 0.14; *p* = 0.61), and CE (r = 0.25 *p* = 0.34). Similarly, a comparison of amino acid solubility of diets within SE, CE, and *in vivo* was performed (Table 5). When correlated within *in vivo*, a significant positive correlation (r = 0.98; *p* < 0.01) was found between reference diet and test diet. Similar correlation (r = 0.81; *p* < 0.01) was found between reference diet and test diet when SE was used. However, a weak but statistically significant correlation (r = 0.54; *p* = 0.03) was observed between the two diets when CE was used.

**TABLE 5:** Correlation between *In vivo*, and *in vitro* protein and amino acid solubility of black soldier fly (BSF) larvae meal, test diet and reference diet using salmon enzymes, and commercial enzymes.

	Correlation between *in vivo* and *in vitro* solubility		
	*In vivo*: *In vitro* SE	*In vivo*: *In vitro* CE	*In vitro* SE: *In vitro* CE
Protein solubility			
Black soldier fly larvae meal	0.989 (0.09)	0.74 (0.47)	0.83 (0.37)
Amino acid solubility			
Black soldier fly larvae meal	0.50 (0.07)	0.19 (0.46)	0.44 (0.09)
Test diet	0.07 (0.79)	0.23 (0.4)	0.27 (0.32)
References diet	0.14 (0.61)	0.25 (0.34)	0.99 (<0.01)

Correlation values (r) are indicated before the parenthesis; *p* values are indicated inside the parenthesis; SE: salmon enzyme; CE: commercial enzyme.

## Discussion

### Standardisation of salmon extracted enzyme and commercial enzyme using pH stat method for *in vitro* solubility and comparison of enzyme efficiency between salmon extracted enzyme and commercial enzyme

The current study used enzyme extracted from salmon gut, and commercial proteases (bovine or porcine) to get an insight about the of degree of hydrolysis (DH%) upon dilution from a fixed concentration apart from the established enzyme concentration. The major goal of diluting the SE and CE was to determine the efficiency of enzyme required to have a comparable hydrolytic capability. According to the findings of this study, when standardised based on DH%, the SE, and CE exhibits similar protein hydrolytic performance. This is because, DH% quantifies the peptides that were cleaved by the amount of enzyme present in the reaction mixture. So, when the volume that exhibited similar DH% were selected, it indirectly quantifies the enzyme required to cleave the peptides. Hence, at this point, the volume selected will have a fixed quantity of enzyme to cleave the fixed substrate. This explains that SE, and CE could behave same once standardised based on DH%. This is different from the enzyme standardisation based on enzyme units which shows significant difference in the protein solubility even after being fixed to same enzyme units (Alarcón et al., 2002). This is primarily due to the differences in the enzyme kinetics, and catalytic properties shown between them. When the enzyme activity of the standardised volume was performed, the salmon pepsin exhibited higher enzyme activity than the equivalent volume of CE that was required to produce the same DH%. This agrees with the study conducted by Norris and Elam, (1940), where the pepsin activity of SE was higher than the porcine pepsin. This might be because cold adapted enzymes have higher catalytic efficiency due to a flexible tertiary structure that helps in lowering the activation energy during catalysis (Hochachka, 1984; Outzen et al., 1996). It can also be speculated that higher pepsin activity might be due to the feeding habit of salmon as pepsin activity is apparently related to predation and carnivorous have highest pepsin level than the herbivores (Einarsson, 1993). Whereas salmon intestinal proteases exhibited similar enzyme activities with CE, as most of the studies suggested that the fish serine proteases are similar to the homeotherms in terms of their molecular size, amino acid composition, and sensitivity to protease inhibitors (Dimes and Haard, 1994; Shahidi and Janak Kamil, 2001).

Thus, it should be noted that even though the enzymes are standardised based on their hydrolytic capacity, the enzyme activity of SE and CE varies because of specific features shown by the enzymes from cold adapted species and carnivorous compared to their counterparts from homeotherms and herbivores/omnivores (Smalås et al., 1994). Therefore, on one hand species specific enzymes extracts are always considered better when performing *in vitro* solubility for feed ingredients, and diets as many other factors such as age, habitat, feeding status, and enzyme properties of the species can also alter the solubility values (Yasumaru and Lemos, 2014). On the other hand, the use of purified commercial enzymes is helpful since it allows the standardisation of *in vitro* digestion models and lab comparisons (Santos et al., 2019).

### *In vitro* solubility of black soldier fly larvae meal and experimental diets using salmon extracted enzyme and commercial enzyme

The DH% is a quantitative measurement of the protein solubility and do not determine the solubility of individual amino acid. The standardised volume of enzyme was used further for determining the amino acid solubility of BSF larvae meal and diets. The two-stage hydrolysis helps in performing the *in vitro* digestion in a more realistic condition than single step hydrolysis (Yasumaru and Lemos, 2014). Moreover, gastric phase including HCl, and pepsin helps in breaking down the peptide bond and leads to higher protein hydrolysis and provides more homogenous solubility values (Alarcón et al., 2002) than single step hydrolysis. The conditions such as temperature, pH, and reaction time selected for the *in vitro* solubility in this study were based on the previous studies (Eggum et al., 1989; Rahmah et al., 2016; Silva et al., 2022). The products after second step of hydrolysis were recovered to evaluate the amino acid solubility of BSF larvae meal and diets. In general, *in vitro* solubility values range obtained for the BSF meal used in this study was similar to the solubility range obtained by Moyano & Savoie (2001) for ingredients like fish meal that showed 20–50% using purified commercial enzymes, and 5–20% using fish digestive extracts. However, low solubility of methionine in this study was observed probably due to the lesser availability of methionine in the BSF larvae meal (Makkar et al., 2014). The reduced solubility of amino acids in the BSF larvae meal using the SE might be associated with the chitin present in the BSF larvae meal which can negatively affect the protein digestibility (Marono et al., 2015; Eggink et al., 2022). Further, the level of lipids can affect the DH and amino acid solubility of BSF larvae meal. Lauric acid and linoleic acid are the most common saturated, and unsaturated lipid fraction present in the BSF larvae meal. Extraction of these lipids can concentrate the proteins and amino acids. This eventually increases the degree of hydrolysis and expose more ionizable amino and carboxylic groups for enzyme hydrolysis (Zayas et al., 1997).

*In vitro* solubility of the experimental diets used in this study was similar to the results obtained by Carter et al. (1999), where the digestibility values ranged from 74 to 89%, and 82–91% using salmon crude enzymes and commercial enzyme, respectively. Furthermore, the results from the study by Rahmah et al. (2020) showed that CE exhibited higher solubility in the experimental diets than the crude intestinal extract of bagrid catfish (*Mystus nemurus*). It can be speculated that this might be due to the combination ratio of various enzymes in the CE mixture or may be because of the higher thermostability of CE than SE (Smalås et al., 1994; Outzen et al., 1996). When compared between the two diets used in the study irrespective of the enzyme source, the amino acid solubility of reference diet and test diet presented similar values indicating that inclusion of insect meal does not affect the overall solubility as reflected *in vivo*. However, the lesser solubilities of lysine, phenylalanine, and tyrosine in test diet with SE could be possibly due to the limited accessibility of amino acid residues in the diet specific to serine proteases chymotrypsin, and trypsin (Alarcón et al., 2002). The other possible reason might be because of the interference of chitin present in the BSF that can prevent the protein break down from the non-soluble fraction. In this case, during hydrolysis, these amino acids might be less accessible for enzymatic breakdown due to aggregation (Slizyte et al., 2005). However, chemical treatment or enzymatic hydrolysis can be used to convert the chitin into more soluble products, and consequently increase the DH and solubility of amino acids.

### *In vivo* digestibility of black soldier fly larvae meal and experimental diets and comparison between *in vitro* and *in vivo*

In the *in vivo* study, BSF larvae meal was found to be highly digestible by Atlantic salmon exhibiting the true protein digestibility around 99%. Similar results were obtained in digestibility studies where the ADC of protein was above 85% and almost 90% in rainbow trout, and Atlantic salmon, respectively using BSF larvae meals (Belghit et al., 2019a; Fisher et al., 2020). The *in vivo* amino acid digestibility values were also similar with the other digestibility trials performed on rainbow trout (Dumas et al., 2018). Similarly, the ADC of diets were comparable with other digestibility studies (Magalhães et al., 2017; Renna et al., 2017). The higher digestibility of BSF larvae meal suggests that it could be used as a potential ingredient in low fish meal and high plant-based protein diet. Furthermore, the main purpose of *in vitro* solubility was to find out the reliability of this method in predicting the digestibility values *in vivo*. A strong correlation between the reference diet and test diet within the *in vivo* and *in vitro* using SE suggests that the inclusion of BSF larvae meal in the diet of Atlantic salmon are equally digested. A strong positive correlation between SE, and *in vivo* was observed for protein solubility in BSF larvae meal suggesting that species specific enzyme extract gives a better reflection of the *in vivo* studies (Alarcón et al., 2002). The enzyme complex extracted from salmon cleaves the peptide bond at the specific sites as *in vivo*. This agrees with many studies that have established a significant correlation between the *in vitro* and *in vivo* methods using fish enzyme extract on protein solubility (Alarcón et al., 2002; Yasumaru and Lemos, 2014; Rahmah et al., 2016; Rahmah et al., 2020).

However, in the current study, an attempt was made to correlate amino acid solubility between *in vivo* and *in vitro*, but no strong correlation was found. The possible reason for lacking correlation between *in vivo* and *in vitro* amino acid solubility data might be due to the following reasons; firstly, DH% that was used to standardise the enzymes, quantified the number of peptide bonds hydrolysed in a given protein source. This eliminates the detection of pre-hydrolysed peptides already present in the reaction mixture. Thus, these undetected amino acids, when analysed will interfere with the final solubility values. Secondly, the differences might be because of the site of hydrolysis by the proteases. This can be related to the specificity of enzymes in cleaving the peptide bond within (endoproteases) or at the end (exoproteases) of protein molecules (Alarcón et al., 2002). Hydrolysis by endo- or exo-proteases by different enzyme sources also depends on the assay conditions. These include factors like the assay temperature, biological age of fish, salinity of the rearing water, particle size of ingredients and diets (Lu et al., 2011). Furthermore, the method cannot reproduce the complex digestion process which includes physical contraction, fluid mechanics of mixing and gradual emptying of food particles, as well as the contribution by the gut microbiota. However, these factors were not considered in this study, and might possibly explain the differences in the relative amino acid solubility values between *in vitro* and *in vivo*.

## Conclusion

Both *in vivo* and *in vitro* experiments revealed that black soldier fly larvae meal as a fish feed ingredient is highly digestible. Data from the present study showed that the enzyme standardised based on hydrolytic capacity using pH stat method can be used to compare and standardise between different source of enzymes. A strong correlation between *in vitro*, and *in vivo* protein solubilities might not necessarily reflect the amino acid solubilities mainly because of the specificity and catalytic properties of enzymes from varied sources. Therefore, species specific enzymes extracts are always better in correlating *in vitro* to *in vivo*. Since fish encompasses a vast number of species, it is difficult to follow the human approach to standardise the operating conditions. As a result, further research focusing on dynamic digestion models using purified fish enzymes will be more suitable in evaluating nutritional digestibility to ensure consistent and reproducible results.

## Data Availability

The original contributions presented in the study are included in the article/Supplementary Material, further inquiries can be directed to the corresponding author.
