# Supplementary material for: Assessing amino acid solubility of black soldier fly larvae meal in Atlantic salmon (Salmo salar) in vivo and in vitro
Source: Front Physiol. 2022 Nov 23;13:1028992. doi: 10.3389/fphys.2022.1028992 (PMC9727232; doi:10.3389/fphys.2022.1028992)
Supplement: Supplementary file 1 [file Table1.DOCX]

Supplementary Material

# Supplementary Tables

**Table 1: *In vitro* amino acid solubility of reference diet and test diet using salmon enzymes, and commercial enzymes after two stage hydrolysis**

|  | ***In vitro* amino acid solubility** | | | | | | |
| --- | --- | --- | --- | --- | --- | --- | --- |
|  | **Salmon enzyme** | | **Commercial enzyme** | | **Two-way ANOVA P-value** | | |
| **Amino acids** | **Reference diet** | **Test diet** | **Reference diet** | **Test diet** | **Enzyme** | **Diet** | **Diet*Enzyme** |
| His | 63.9±1.5 | 58.7±3.5 | 70.2±0.3 | 69.9±0.09 | 0.003 | ns | ns |
| Ser | 65.4±0.0 | 61.6±3.4 | 66.3±1.4 | 68.4±0.4 | 0.04 | ns | ns |
| Arg | 67.9±1.7 | 64.1±3.3 | 75.2±0.6 | 75.1±0.7 | 0.002 | ns | ns |
| Gly | 68.7±0.9 | 65.1±3.3 | 69.1±1.4 | 70.0±0.9 | Ns | ns | ns |
| Asp | 65.4±1.3 | 58.9±4.1 | 70.4±1.0^b^ | 90.6±0.7^a^ | <0.001 | 0.012 | <0.001 |
| Glu | 76.1±1.0 | 70.3±2.9 | 80.9±0.5 ^b^ | 91.9±0.3 ^a^ | <0.001 | ns | <0.001 |
| Thr | 63.8±2.1 | 59.1±0.4 | 66.0±1.2 | 66.6±1.1 | 0.04 | ns | ns |
| Ala | 66.0±1.4 | 62.9±3.7 | 67.9±1.4 | 70.5±0.1 | 0.03 | ns | ns |
| Pro | 73.0±1.3 | 69.4±3.0 | 75.4±1.0 | 73.6±2.8 | Ns | ns | ns |
| Lys | 67.6±0.9 | 54.8±0.3 | 73.3±1.4 | 80.9± 12.0 | 0.02 | ns | ns |
| Tyr | 63.0±1.9^a^ | 48.4±5.9^b^ | 66.4±0.9 | 64.6±0.0 | 0.01 | 0.021 | 0.05 |
| Met | 61.1±2.1 | 58.2±4.5 | 64.2±1.4 | 69.4±1.7 | 0.02 | ns | ns |
| Val | 62.8±0.7^a^ | 49.5±0.05^b^ | 64.3±1.4^b^ | 77.9±0.8^a^ | <0.001 | ns | <0.001 |
| Ile | 62.9±1.6 | 54.2±6.2 | 65.2±1.6^b^ | 82.5±0.1^a^ | 0.002 | ns | 0.005 |
| Leu | 65.7±1.3^a^ | 61.2±0.6^b^ | 69.2±0.9 | 69.9±1.5 | 0.001 | ns | 0.031 |
| Phe | 61.7±1.5^a^ | 49.9±0.06^b^ | 66.5±0.8 | 64.8±1.3 | <0.001 | <0.001 | 0.003 |

All data are shown as mean±SD (n=2). P values of two-way ANOVA are presented for factors ‘diet’, ‘enzyme’ and interaction between diet and enzyme. ns, not statistically significant (p > 0.05). Different superscript letters within an individual row denote statistically significant differences in amino acid solubility according to Tukey’s multiple comparison test

**Table 2: *In vitro* amino acid solubility of black soldier fly (BSF) larvae meal using salmon enzyme, and commercial enzyme after two stage hydrolysis**

| Amino acid | Salmon enzyme | Commercial enzyme | P value |
| --- | --- | --- | --- |
| His | 10.5±5.6 | 17.2±1.3 | ns |
| Ser | 18.9±7.5 | 25.6±0.6 | ns |
| Arg | 19.6±6.2^b^ | 35.4±0.3^a^ | 0.046 |
| Gly | 38.6±15.3 | 27.4±0.6 | ns |
| Asp | 14.5±6.3 | 23.9±0.02 | ns |
| Glu | 22.4±5.3^b^ | 35.5±0.4^a^ | 0.038 |
| Thr | 15.3±6.2 | 24.4±0.2 | ns |
| Ala | 44.5±17.8 | 51.0±24.1 | ns |
| Pro | 28.9±8.5^b^ | 37.6±1.3^a^ | 0.023 |
| Lys | 22.7±5.9 | 35.9±1.7 | ns |
| Tyr | 36.9±13.1^a^ | 17.9±2.6^b^ | 0.043 |
| Met | 5.3±7.9 | 15.1±0.06 | ns |
| Val | 4.0±4.0^b^ | 27.4±3.8^a^ | 0.046 |
| Ile | 28.2±0.5^b^ | 41.7±9.9 ^a^ | 0.015 |
| Leu | 44.5±12.7^b^ | 39.3±0.4 ^a^ | 0.014 |
| Phe | 34.6±5.4^b^ | 23.4±4.8 ^a^ | 0.009 |

The values are expressed as mean±SD (n=3). Small letters (a, and b) indicate the statistical difference between salmon enzyme, and commercial enzyme detected with one-way ANOVA followed by Tukey’s multiple comparisons

**Table 3. Apparent digestibility coefficient (ADC%) of crude lipid, true protein, crude protein, and amino acid of black soldier fly larvae meal (BSF)**

| **Nutrients** | **ADC% of BSF larvae meal** |
| --- | --- |
| Crude lipid | 68.9±4.4 |
| True protein | 99.18±7.3 |
| Crude protein | 89.9±5.4 |
| **Amino acids** | |
| His | 112.8±3.9 |
| Ser | 94.8±6.9 |
| Arg | 92.0±3.7 |
| Gly | 90.9±8.6 |
| Asp | 69.7±10.0 |
| Glu | 108.1±6.6 |
| Thr | 93.5±6.8 |
| Ala | 99.6±5.1 |
| Pro | 100.4±5.7 |
| Lys | 104.2±4.2 |
| Tyr | 102.9±2.7 |
| Met | 80.0±6.7 |
| Val | 91.6±5.1 |
| Ile | 87.9±4.9 |
| Leu | 101.8±4.9 |
| Phe | 94.3±4.5 |

The values are expressed as mean±SD (n=3).

**
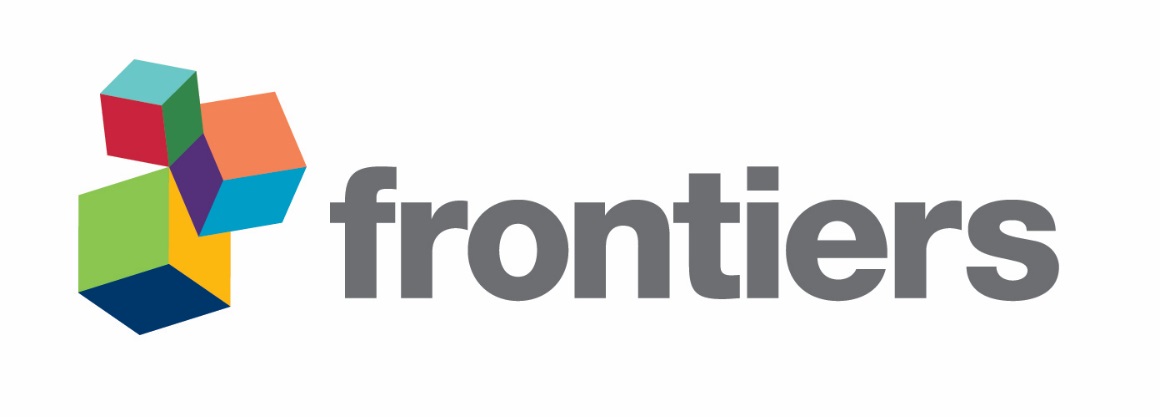
**
